# Patient-Derived Organoid Models of Human Neuroendocrine Carcinoma

**DOI:** 10.3389/fendo.2021.627819

**Published:** 2021-03-11

**Authors:** Krijn K. Dijkstra, José G. van den Berg, Fleur Weeber, Joris van de Haar, Arno Velds, Sovann Kaing, Dennis D. G. C. Peters, Ferry A. L. M. Eskens, Derk-Jan A. de Groot, Margot E. T. Tesselaar, Emile E. Voest

**Affiliations:** ^1^ Department of Molecular Oncology and Immunology, Netherlands Cancer Institute, Amsterdam, Netherlands; ^2^ Oncode Institute, Amsterdam, Netherlands; ^3^ Department of Pathology, Netherlands Cancer Institute, Amsterdam, Netherlands; ^4^ Central Genomics Facility, Netherlands Cancer Institute, Amsterdam, Netherlands; ^5^ Core Facility Molecular Pathology and Biobanking, Netherlands Cancer Institute, Amsterdam, Netherlands; ^6^ Department of Medical Oncology, Erasmus Medical Center, Rotterdam, Netherlands; ^7^ Department of Medical Oncology, University Medical Center Groningen, Groningen, Netherlands; ^8^ Department of Medical Oncology, Netherlands Cancer Institute, Amsterdam, Netherlands

**Keywords:** gastroenteropancreatic neuroendocrine carcinoma (GEP-NEC), extrapulmonary neuroendocrine carcinoma, disease modeling, pre-clinical models, organoids

## Abstract

Gastroenteropancreatic neuroendocrine carcinoma (GEP-NEC) is a poorly understood disease with limited treatment options. A better understanding of this disease would greatly benefit from the availability of representative preclinical models. Here, we present the potential of tumor organoids, three-dimensional cultures of tumor cells, to model GEP-NEC. We established three GEP-NEC organoid lines, originating from the stomach and colon, and characterized them using DNA sequencing and immunohistochemistry. Organoids largely resembled the original tumor in expression of synaptophysin, chromogranin and Ki-67. Models derived from tumors containing both neuroendocrine and non-neuroendocrine components were at risk of overgrowth by non-neuroendocrine tumor cells. Organoids were derived from patients treated with cisplatin and everolimus and for the three patients studied, organoid chemosensitivity paralleled clinical response. We demonstrate the feasibility of establishing NEC organoid lines and their potential applications. Organoid culture has the potential to greatly extend the repertoire of preclinical models for GEP-NEC, supporting drug development for this difficult-to-treat tumor type.

## Introduction

NEC are poorly differentiated, fast-growing tumors that arise in various tissues, with a median survival of 5 months for metastatic disease (1, 2). Most extrapulmonary NEC arise in the gastroenteropancreatic tract (GEP-NEC) (3, 4). Patients with GEP-NEC often present with advanced disease at diagnosis (4, 5) and treatment options are limited. First-line treatment for patients with metastatic GEP-NEC consists of combination platinum-based chemotherapy. Response rate is relatively high (42%–67%), but patients relapse rapidly (5). No standard second-line therapy has been established.

Due to its low incidence (3, 4), GEP-NEC is a relatively understudied disease. Genetic characterization is limited to small studies and targeted sequencing of a few cancer genes (6–8). Because of its low incidence, accrual into clinical trials and development of new treatments is slow. There is an unmet need for better preclinical models to increase understanding of this disease and accelerate drug development. However, only few GEP-NEC cell lines have been established, the majority of which are derived from colorectal NEC (9).

Tumor organoids are three-dimensional cultures of tumor cells that retain histomorphologic and genetic features of the original tumor and can be established with high success rate from various tumors (10). Organoid models have been established from four patients with metastatic neuroendocrine prostate cancer (11) two colorectal NEC (12) and a NEC originating from the ampulla of Vater (13). In addition, recently an organoid biobank of neuroendocrine neoplasms has been established (14). Encouraged by these reports, we set out to systematically evaluate the feasibility of establishing GEP-NEC organoid lines, as well as their potential applications. Here, we established three organoid lines of NEC originating from the stomach and colon, describe their histomorphological, immunohistochemical, and genetic properties and drug response. Organoid models could greatly expand the repertoire of pre—clinical models available for the study of GEP-NEC.

## Materials and Methods

### Patient Tissue

Tissue was obtained from patients with metastatic or unresectable neuroendocrine carcinoma of extrapulmonary origin, being treated with everolimus and cisplatin in a phase II clinical trial (clinicaltrials.gov identifier NCT02695459). The study was approved by the Medical Ethical Committee of the Netherlands Cancer Institute – Antoni van Leeuwenhoek and written informed consent was obtained from all patients. Patients were treated in cycles of 75 mg/m^2^ intravenous cisplatinum on day 1 and 7.5 mg everolimus daily orally on days 1–21. Prior to treatment, a 18G core needle biopsy of the tumor was taken and processed for organoid culture within 24 h.

### Organoid Establishment and Culture

Organoids were established based on the protocol for colorectal cancer (CRC) organoids (15). Tumor tissue was dissociated into small pieces using razor blades or needles and enzymatically digested using 1.5 mg/ml collagenase type II (Sigma-Aldrich), 10 µg/ml hyaluronidase type IV (Sigma-Aldrich), 10 µM Y-27632 (Sigma-Aldrich) and 1:500 Primocin (Invivogen) at 37°C for 30 min. After washing, tumor cells were embedded in 10 mg/ml lactate dehydrogenase elevating virus (LDEV)-free reduced growth factor basement membrane extract (Gibco) and plated in small drops on a six-well culture plate. Geltrex drops were solidified for 20 min at 37°C before overlaying them with NEC organoid medium. NEC organoid medium consisted of Advanced DMEM/F12 (GIBCO) supplemented with 2 mM Ultraglutamine I (Lonza), 10 mM HEPES (GIBCO), and 100/100 U/ml Pencillin/Streptomycin (GIBCO), 50% Wnt-3a-conditioned medium (CM), 10% Noggin-CM, 20% R-spondin1-CM, 1x B27 supplement without vitamin A (GIBCO), 1.25 mM N-Acetylcysteine (Sigma-Aldrich), 10 mM nicotinamide (Sigma-Aldrich), 50 ng/ml human recombinant EGF (Peprotech), 100 ng/ml IGF-1 (Peprotech), 500 nM A83-01 (Tocris), 10 µM SB202190 (Cayman Chemicals), 10 µM Y-27632, 10 nM gastrin (Sigma-Aldrich) and 10 nM prostaglandin E2 (Cayman Chemicals). Until cryopreservation, organoids were cultured in an incubator at 37°C, under normoxic conditions with 5% CO_2_, in the presence of 1:500 Primocin. Medium was refreshed every 3–4 days and organoids were passaged every 1–2 weeks at a 1:2 to 1:5 split ratio. For passaging, medium was removed, organoids were resuspended in 1 ml/well dispase type II and incubated for 5 min at 37°C. Organoids were collected in 10 ml PBS and 100 µl of 0.5 M EDTA was added for every 1 ml of dispase used. After washing, organoids were resuspended in 1–4 ml accutase (Stem Cell Technologies) and incubated for 5–10 min at 37°C. Organoids were washed in PBS and replated in Geltrex drops. Organoids were cryopreserved in Recovery Cell Culture Recovery Freezing Medium (Gibco) or 10% DMSO/FCS at −80°C or in liquid nitrogen.

Some lines were cultured in tissue-specific medium rather than the generic medium described above (**Supplementary Table 1**). Medium formulation was based on previously published reports (pancreas (16), ovarium (17), stomach (18), colorectal (15)).

Organoids were authenticated by single nucleotide polymorphism (SNP) array, short tandem repeat (STR) profiling or human leukocyte antigen (HLA) haplotyping. For SNParray, organoids and matching blood were typed for 26 single nucleotide polymorphisms (SNPs) using a Taqman-based SNParray (Hartwig Medical Foundation, Amsterdam). For STR profiling, samples were matches using a PowerPlex 16 HS System (Promega, DC2101). HLA-A, -B, -DRB1 low-intermediate resolution typing was performed at the dept. of Sanquin HLA diagnostic services by means of the Sequence Specific Oligonucleotide probes (SSOP) typing method using the Luminex microbead technology which involved polymerase chain reaction (PCR) amplification of the HLA class I and II regions (Lifecodes HLA typing kits, Genprobe, San Diego CA-USA). All samples were regularly tested for Mycoplasma using the MycoAlert Mycoplasma Detection Kit (Lonza).

### Immunohistochemistry and Imaging

Organoids were isolated from Geltrex by incubating in 2 mg/ml dispase for 5 min at 37°C, followed by washing in 10 ml 5 mM EDTA/PBS. In some cases, dispase treatment resulted in organoid dissociation. Therefore we resupended organoids in ice-cold PBS to isolate organoids from Geltrex in some cases. Organoids were pelleted and fixed in 4% paraformaldehyde for 30 min at room temperature, or for 24 h at 4°C. After fixation, organoids were washed in PBS and embedded in paraffin blocks. 3 µm paraffin sections were cut and routine hematoxylin and eosin (H&E) staining was performed.

Immunohistochemistry of the formalin-fixed, paraffin-embedded (FFPE) tumor samples was performed on a BenchMark or Discovery Ultra autostainer (Ventana Medical Systems). Briefly, paraffin sections were cut at 3 µm, heated at 75°C for 28 min and deparaffinized in the instrument with EZ prep solution (Ventana Medical Systems). Heat-induced antigen retrieval was carried out using Cell Conditioning 1 (CC1, Ventana Medical Systems) for 32 min at 95°C (Synaptophysin, Chromogranin, MLH1, MSH2, MSH6, CD56), 64 min at 95°C (Ki-67) or 72 min at 95°C (PMS2). The following primary antibodies were used: chromogranin was detected using clone LK2H10 (Ready-to-Use, 32 min at 37°C, Roche), Synaptophysin using clone 27G12 (Leica) 1/50 dilution for 32 min at 37°C, Ki-67 using clone MIB1 (1/100 dilution, 1 hour at 37°C, Agilent/DAKO), MLH1 using clone ES05 (1/20 dilution, 32 min at 37°C, Agilent/DAKO), MSH2 using clone G219-1129 (Ready-To-Use, 12 min at 37°C, Roche/Ventana), MSH6 using clone EP49 (1/50 dilution, 32 min at 37°C, Epitomics), PMS2 using clone A16-4 (Ready-To-Use, 32 min at 37°C, Roche/Ventana), and CD56 using Clone MRQ-42 (Ready-To-Use, 4 min at 37°C, Ventana Medical Systems).

To reduce background staining, after incubation of the primary antibodies chromogranin A, synaptophysin and Ki67, a rabbit anti-mouse IgG1, IgG2a, IgG3 (Clone M204-3, Abcam, ab1334698, 1/500, 32 min at 37°C) was added, after which bound antibody for chromogranin A, synaptophysin, Ki67 and CD56 was visualized using Anti-Rabbit HQ (Ready to Use, 12 min at 37°C, Ventana Medical Systems), Anti-HQ HRP (Ready to Use, 12 min at 37°C, Ventana Medical Systems) and ChromoMap DAB (Ventana Medical Systems). For negative controls, stainings were performed without the primary antibody. For MLH1 and PMS2 signal amplification was applied using the Optiview Amplification Kit (4 min, Ventana Medical Systems). For MLH1, MSH2, MSH6 and PMS2 bound antibody was visualized using the OptiView DAB Detection Kit (Ventana Medical Systems). Slides were counterstained with Hematoxylin and Bluing Reagent (Ventana Medical Systems).

### Drug Assays

Organoids were isolated from Geltrex 4 days after passaging by incubating in 2 mg/ml dispase type II for 5 min at 37°C, followed by washing in 5 mM EDTA/PBS. Organoids were counted and resuspended in 10 mg/ml Geltrex at 50 organoids/µl. Five-microliter drops were plated in pre-warmed clear-bottom white-walled flat-bottom 96-well culture plates (Greiner). Geltrex drops were allowed to solidify at 37°C for 15 min before overlaying with 200 µl/well of drug-containing NEC organoid medium. Five-step ten-fold serial dilutions were made of cisplatin (50 µg/ml to 5 ng/ml, PBS, Accord Health Care, Netherlands) and everolimus (25 µM to 250 fM, DMSO, Selleck Chemicals). For untreated controls, the volume of DMSO corresponding to the highest drug concentration was added to NEC organoid medium. After 6 days culture in the presence or absence of drugs, viability was evaluated using CellTiter-Glo 3D (Promega) on an Infinite 200 Pro plate reader (Tecan Life Sciences). Medium was replaced by 50 µl Ad-DF+++ mixed with 50 µl CellTiter-Glo reagent, incubated in the dark for 5 min on an orbital shaker (room temperature), followed by 25 min incubation without shaking. Baseline measurements were taken at the time of plating (day 0).

Growth rate (GR) corrected viability was calculated as described before (19)

$$GR=2^{\frac{log\text{ 2(}\frac{Vd}{Vo})}{log2(\frac{Vctrl}{V0})}}-1$$

where V_d_ is the viability of drug-treated organoids at day 6, V_0_ is median viability of baseline measurement (day 0), and V_ctrl_ is the viability of control-treated organoids at day 6 (based on CellTiter Glo). Data was analyzed using GraphPad Prism V7.03. Dose response curves were fit on data normalized to control-treated samples using Prism based with a hill slope of −1, and top constrained to 100. IC_50_ or GR_0_ values are absolute and interpolated from dose response curves.

### DNA Sequencing

DNA was isolated from organoids and patient-matched blood using a DNEasy blood and tissue kit (Qiagen). At least 200 ng of DNA was used for whole-exome sequencing after exome capture (Integrated DNA Technologies probe set) and sequenced on an Illumina NextSeq DNA analyzer using 150 base pair paired-end reads. Reads were aligned to a reference genome (GRCh38) with BWA-MEM (20), tagged, if duplicate, by Picard MarkDuplicates, and base quality score normalized using GATK BaseRecalibrator (GATK version 4.1) (21). Hereafter somatic and germline single nucleotide variants (SNVs) were called by GATK MuTect 2 and HaplotypeCaller, respectively. Copy number, cellularity and ploidy were estimated, from the paired tumor-normal samples, and visualized using Sequenza (22).

Somatic mutations were annotated using SnpEff (23) and SnpSift (24) was used to remove silent and non-coding mutations. Specifically, retained mutations comprised those annotated as high or moderate impact, and/or having an effect of the following categories: ‘coding_sequence_variant’, ‘disruptive_inframe_deletion’, ‘disruptive_inframe_insertion’, ‘exon_variant’, ‘feature_ablation’, ‘frameshift_variant’, ‘gene_variant’, ‘inframe_deletion’, ‘inframe_insertion’, ‘missense_variant’, ‘protein_protein_contact’, ‘rare_amino_acid_variant’, ‘stop_gained’, ‘stop_lost’, ‘structural_interaction_variant’, ‘transcript_variant’. Mutated genes were filtered for cancer genes, using the MSK-IMPACT cancer gene panel (25).

In addition, DNA was used for low-coverage whole genome sequencing for copy number analysis on an Illumina HiSeq2500 DNA analyzer (High Output Mode) using 65 base pair single reads. Reads were aligned to GRCh38 using BWA-MEM (20). Per sample, per bin of 20 kb, mapping quality 37 read counts were rated against mappability, predicted by a likewise mapping of commensurate GRCh38 reference derived sequences, and log2 transformed. Sample counts were corrected per bin for local GC effects using a non-linear fit of mappabilities over 0.8 on autosomes. Reference values were scaled according to the slope of a linear fit of reference mappabilities after GC correction. Ratios of corrected sample counts and reference values left out bins with mappability below 0.2 or overlapping ENCODE blacklisted regions (26). Resulting copy number data were analyzed using Nexus v10.0.

### Statistics

IC_50_ or GR_0_ values were compared to NEC-01013 as a reference using a one-way ANOVA with Dunnett’s multiple comparison test. Other statistics are based on two-sided Student’s t test. P-values < 0.05 are considered statistically significant. The number of asterisks in figures indicates the level of significance: * P < 0.05; ** P < 0.01; *** P < 0.001.

## Results

### Organoid Establishment

We generated GEP-NEC organoid lines using a procedure similar to that established for CRC organoids (15). We initially based our culture medium on that used for CRC organoids, with the addition of IGF-1 based on high expression of phosphorylated mTOR in NEC (27). We used this medium to establish organoids from core needle biopsies from 22 patients with metastatic or inoperable extrapulmonary NEC. From this initial cohort, organoid lines were established from three patients: NEC-01010, derived from a liver metastasis originating from the ampulla of Vater; NEC-01013, derived from a liver metastasis of a gastric NEC; and NEC-02002, derived *via* a colonic biopsy.

As we received NEC from various tissues (**Supplementary Figure 1**), in a second phase we attempted organoid culture using a modified protocol in which the culture medium was adjusted (where possible) for each sample based on its tissue of origin (**Supplementary Table 1**). This resulted in the establishment of two additional organoid lines (NEC-02006, from a liver metastasis of a NEC originating from the colon; and NEC-02007, a gastric NEC). Organoid establishment rates were not significantly different for samples cultured in generic NEC medium (three out of 24 successful) versus tissue-specific medium (two out of 7, Fisher’s exact test: p = 0.31). Overall establishment rate (combining both protocols) was 16% (5 out of 31).

**Figure 1:**
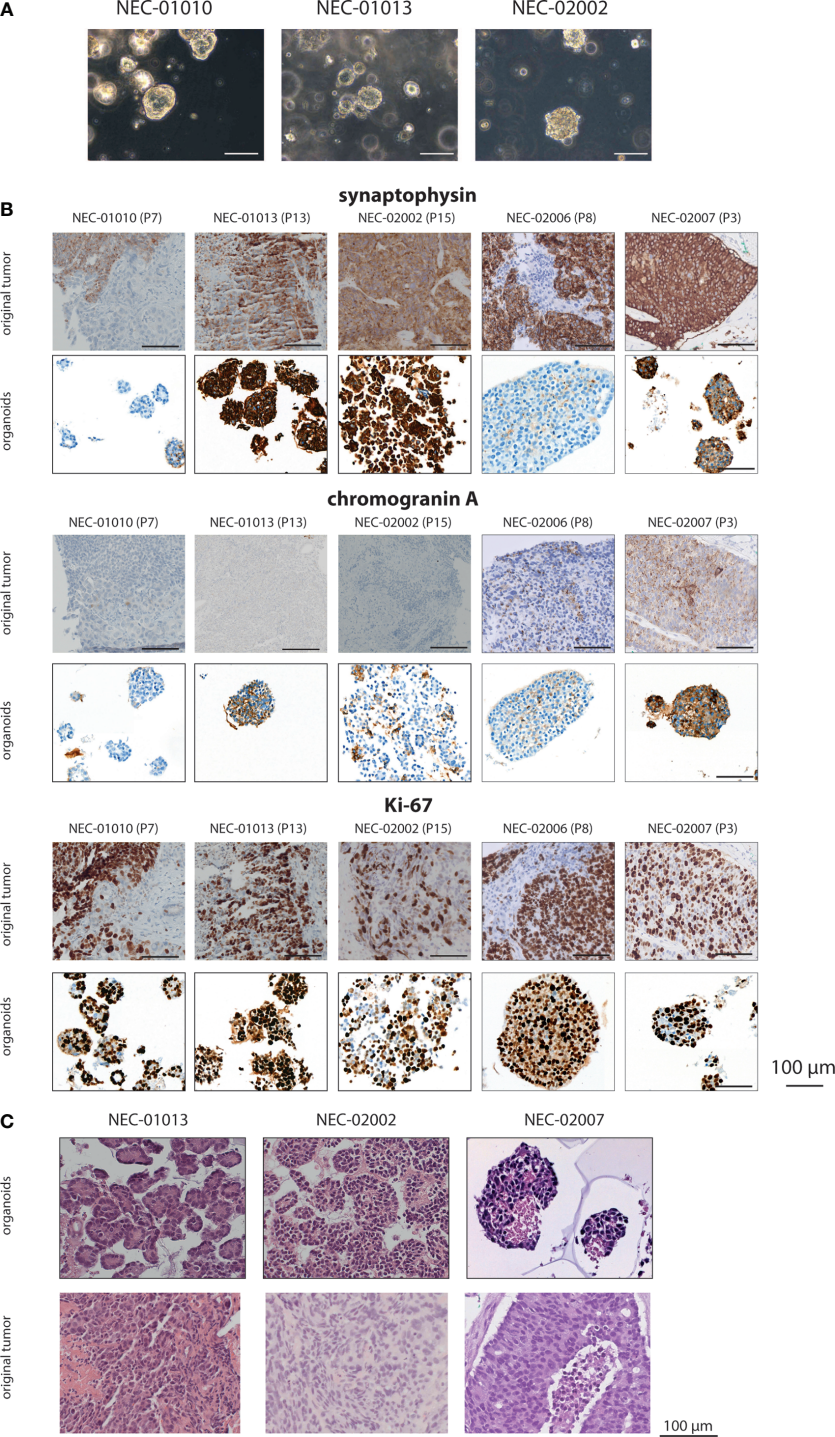
Histomorphology of GEP-NEC organoid lines and matching original tumor. **(A)** Phase-contrast photomicrographs of GEP-NEC organoid lines. Scale bar = 100 µm. **(B)** Immunostainings of synaptophysin, chromogranin and Ki-67 for organoids and original tumor. P7 indicates passage 7. **(C)** Hematoxylin and eosin (H&E) stained slides of GEP-NEC organoid lines and original tumors. NEC-01013: passage (P) 12; NEC-02002: P20; NEC-02007: P3.

Organoids grew as solid, slightly irregular spheres (**Figure 1A**) and could be passaged with a biweekly split ratio of 1:2 to 1:5. After 5–10 weeks of expansion, organoids were frozen, from which they recovered well (only one sample, which we classified as a failed culture, did not survive cryopreservation). Organoids were cultured until passages 17–22, or 7–10 months in culture. We did not observe any decrease in proliferation at higher passage, and cultures were terminated for practical reasons rather than because of ceased growth.

### Immunohistochemical Characterization of Organoid Lines and Original Tumors

To confirm the neuroendocrine nature of the established organoid lines, we evaluated the expression of the neuroendocrine markers synaptophysin and chromogranin (**Figure 1B**, **Supplementary Figure 2A** and **Table 1**).

**Table 1:** Expression of Ki-67 and neuroendocrine markers in original tumor and organoids^1^.

	Synaptophysin		Chromogranin A		Ki-67	
	Original tumor	Organoids	Original tumor	Organoids	Original tumor	Organoids
NEC-01010 (P7)^2^	50%^3^	0%	0%	0%	70%	90%
NEC-01013 (P20)	80%	100%	0%	0%	100%	90%
NEC-02002 (P15)	100%	100%	0%	0%	40%	60%
NEC-02006 (P8)	80%	0%	20%	0%	N/A	90%
NEC-02007 (P3)	100%	50%	80%	60%	70%	70%

^1^Values indicate percentage of cells positive for the marker.

^2^P7 indicates organoids were stained at passage 7.

^3^NEC-01010 showed heterogeneous expression of synaptophysin, with some areas being completely negative, and some areas 100% positive.

Based on these markers, organoid lines NEC-01013, NEC-02002, and NEC-02007 could be reliably classified as NEC organoids. The original tumor and corresponding organoid lines expressed synaptophysin in all three cases. NEC-01013 and NEC-02002 were negative for chromogranin (both in the original tumor and the matching organoid lines), but NEC-02007 showed chromogranin expression in organoids as well as the original tumor. The percentage of cells expressing Ki-67 was comparable between organoids and original tumor (**Table 1**).

In contrast, expression of neuroendocrine makers was discordant between organoids and original tumor for lines NEC-01010 and NEC-02006. The original tumor from NEC-01010 was negative for chromogranin and showed heterogeneous synaptophysin expression (50% overall, varying from absent to strong, both in solid and in glandular parts of the tumor (**Supplementary Figure 2B**). However, organoids were negative for both chromogranin and synaptophysin. Even though 30% of organoids expressed CD56, the original tumor showed only limited CD56 expression, which did not track closely with expression of synaptophysin (**Supplementary Figure 3**). NEC-02006 organoids (a liver metastasis of a NEC originating from the colon) were negative for all NEC markers tested (synaptophysin, chromogranin A and CD56; **Figure 1B** and **Supplementary Figure 3**). Based on the absence of NEC marker expression in NEC-01010 and NEC-02006, we cannot confidently classify these lines as NEC organoids.

Histomorphologically, NEC organoid lines NEC-01013, NEC-02002 and NEC-02007 grew as irregular nests, with large, hyperchromatic nuclei and limited cytoplasm (**Figure 1C**). If only organoids that can reliably be classified as NEC are considered, organoid establishment rate is 10% (3 out of 31).

### Genetic Characterization of Organoid Lines

**Figure 2:**
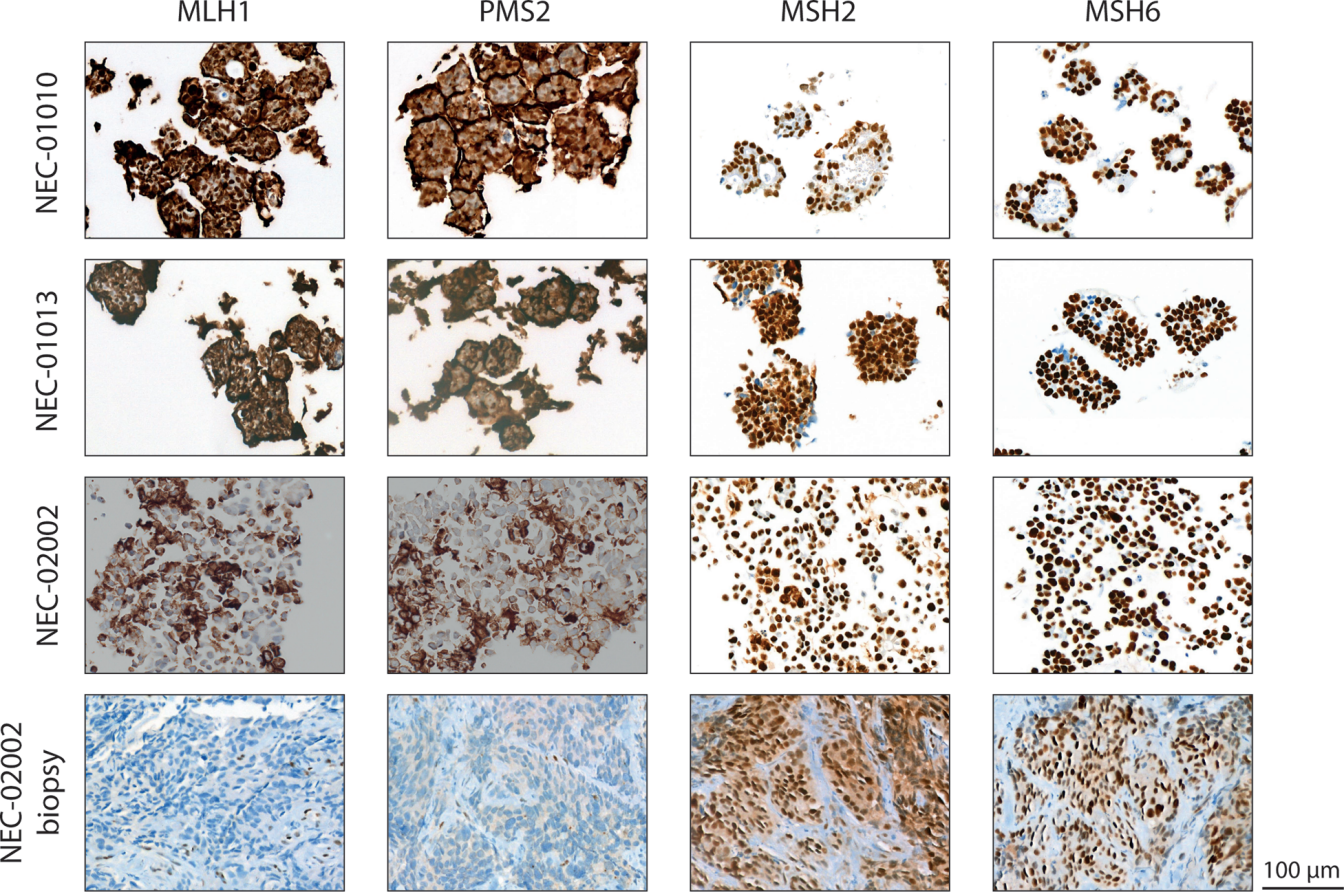
Mismatch repair status of GEP-NEC organoid lines. Staining of slides from NEC-01010 (P7 and P11), NEC-01013 (P13), and NEC-02002 organoids (P15), as well as the original tumor of NEC-02002, for mismatch repair proteins MLH1, PMS2, MSH2, and MSH6. Note 100% nuclear expression of MHS2 and MSH6 but absence of nuclear staining of MLH1 and PMS2 in both organoids and original tumor of NEC-02002. The brown halo on the cell border is a specific staining caused by residual Geltrex in organoid slides. Scale bar = 100 µm.

We characterized organoid lines NEC-01013 and NEC-02002 in more detail. We performed whole exome sequencing (WES) to detect mutations, and low-coverage whole genome sequencing (WGS) for copy number analysis. NEC-01013 and NEC-02002 contained 49 and 532 mutations, respectively (**Table 2**). The high number of mutations in NEC-02002 was paralleled by a relatively high proportion of indels. This genomic profile is characteristic of mismatch repair (MMR) deficiency, and NEC-02002 organoids as well as the original tumor indeed showed loss of expression of the MMR proteins PMS2 and MLH1 (**Figure 2**). Loss of RB1 expression and mutations in *TP53* are frequently found in NEC (6–8, 28). *TP53* mutations were present in both NEC-01013 and NEC-02002 (**Table 2**). NEC-01013 also contained an *RB1* mutation. An *RB1* mutation was also identified in NEC-02002 (**Supplementary Table 2**), but this was an intron variant with low allele frequency and therefore unlikely to be a strong driver. Other mutated cancer genes included those involved in Wnt signaling (*APC*, *AMER1*), mitogen signaling (*KRAS*, *BRAF*), epigenetic modification (*ARID1A*, *ARID2*, *DNMT3A*, *NCOA3*) and DNA repair (*RAD50*, *BRIP1*, *ERCC5*, *RECQL4*).

**Table 2:** Mutations in NEC organoids identified by whole exome sequencing.^4^

	NEC-01013 (P11)	NEC-02002 (P5)
Number of somatic mutations	49	532
Number of indels (of all mutations)	5 (10.2%)	125 (23.5%)
Number of single nucleotide variants (of all mutations)	44 (89.8%)	407 (78.5%)
Mutated cancer genes (VAF)	TP53 (95,2%); KRAS (42,1%); DNMT3A (96,6%); HIST1H3I (45,8%); FLT3 (99,1%); ARID2 (44,2%); NCOA3 (4,7%); RB1 (99%); APC (98,5%); RAD50 (11,1%)	GNA11 (49,4%); TP53 (98,3%); LATS2 (32,8%); DNMT3A (7,6%); CDK8 (21,6%); ARID1A (21,8%); BRIP1 (5,1%); AMER1 (5,5%); ZFHX3 (16,3%); ERCC5 (40,7%); BRAF (35,9%); RECQL4 (15,4%)^5^; RECQL4 (12,7%)

^4^Total number of somatic mutations is described and mutated cancer genes, filtered based on the MSK-IMPACT cancer gene panel, and only including genes with predicted moderate or high impact, are specifically indicated (25).

^5^NEC-02002 contained two different mutations in RECQL4: a frameshift mutation at chr8:144512965 and a missense mutation at chr8:144515376. VAF, variant allele frequency.

**Figure 3:**
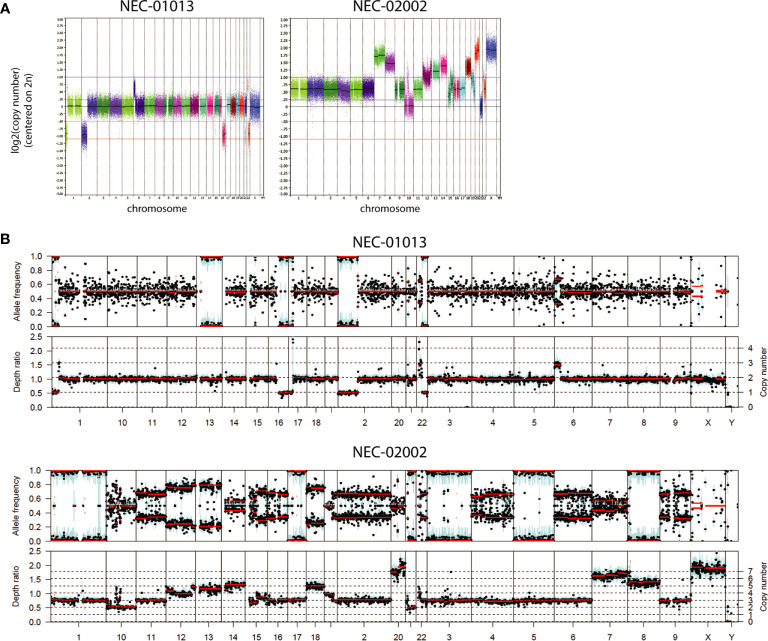
Copy number profiles of GEP-NEC organoids. **(A)** Copy number profiles of GEP-NEC organoids are generated based on low-coverage whole genome sequencing and represented on a log_2_ scale. NEC-01013: passage (P) 11; NEC-02002: P5. **(B)** Copy number profiles were based on SNPs identified by whole exome sequencing and analyzed by Sequenza. Allele frequencies are given as ratios and used to infer copy number. NEC-01013: P11; NEC-02002: P15.

NEC organoids showed variable degrees of chromosomal abnormalities (**Figure 3A** and **Table 3**). NEC-01013 showed a relatively normal genome. In contrast, NEC-02002 showed a highly disorganized genome, with 87% of the genome showing abnormal copy number. We used Sequenza to determine allele-specific copy number based on SNPs identified by WES. This revealed whole-chromosomal losses resulting in loss of heterozygosity (LOH) (**Figure 3B**), further underscoring the level of genomic disorganization of NEC-02002.

**Table 3:** Copy number alterations in NEC organoids.

	NEC-01013 (P11)	NEC-02002 (P5)
% of genome altered	8.4	87.1
Total CN aberrations^6^	64	179
One copy gain	11	108
Two or more copy gain	1	64
One copy loss	33	5
Two copy loss	19	2

^6^CN, copy number.

### Organoid Drug Sensitivity Mirrors Patient Response to Cisplatin and Everolimus

Organoids were derived from patients treated with cisplatin and the mTOR inhibitor everolimus. We therefore determined the response of NEC organoids to these drugs and compared this to patient response for NEC-01010, NEC-01013 and NEC-02002. NEC-01013 was the only patient who showed an objective partial response (PR) after three cycles of chemotherapy. Patient NEC-01010 showed stable disease (SD) after three treatment cycles, but a mixed response at the next evaluation, with some lesions progressing and others regressing (the lesion from which the organoids were derived had not changed in size, but had become more hypodense). Patient NEC-02002 showed limited progression of the liver metastases that still fell within the boundaries of SD at the first two evaluations, and then progressed.

**Figure 4:**
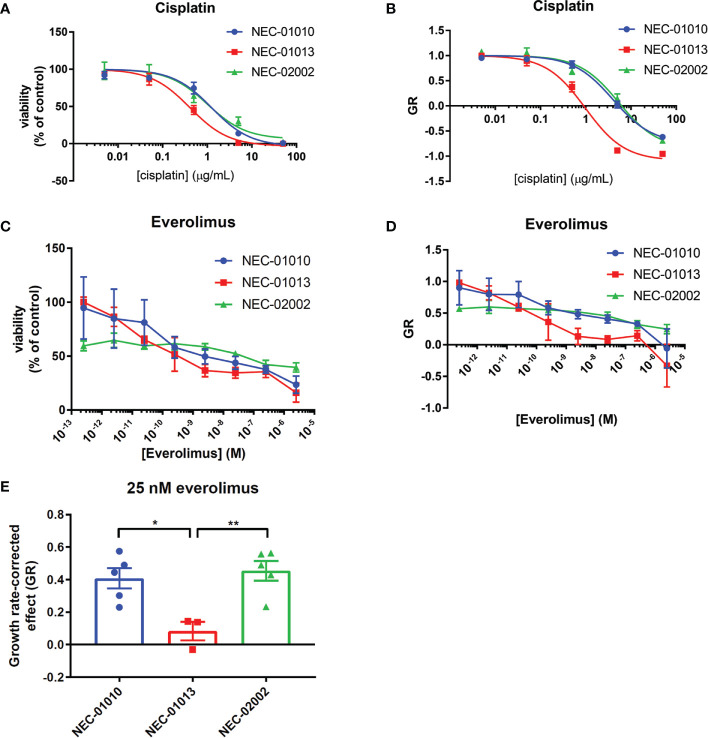
Drug response of GEP-NEC organoids. **(A, B)** Viability **(A)** or growth-rate corrected viability **(B)** of cisplatin-treated GEP-NEC organoids. n = 3–4. NEC-01010: passage (P) 6-14; NEC-01013: P10-19; NEC-02002: P6-16. **(C, D)** Viability **(C)** or growth-rate corrected viability **(D)** of everolimus-treated GEP-NEC organoids. n = 3–5. NEC-01010: P6-16; NEC-01013: P10-20; NEC-02002: P6-17. **(E)** GR at 25 nM everolimus (corresponding to C_max_). n = 3–5. Error bars represent s.e.m. Student’s t test. *P < 0.05; **P < 0.01.

Because the clinical effect of everolimus is often limited to disease stabilization rather than tumor regression (29), we considered two different metrics to determine organoid drug sensitivity. First, we generated dose response curves and calculated IC_50_ (**Figures 4A, C**). We also evaluated the effect of drug treatment on organoid growth rate (**Figures 4B, D**). The growth rate metric (GR) compares the amount of tumor cells between the start and end of the drug assay (6 days), with a value of –1 indicating complete tumor cell death, a value of 0 complete growth inhibition, and a value of 1 no effect of the drug (19).

NEC-01013 (the only patient with a partial response) was more sensitive to cisplatin than NEC-01010 and NEC-02002, showing statistically significant differences in GR_0_ (**Figures 4A, B**, and **Table 4**). Everolimus did not lead to complete tumor cell death, even at concentrations far exceeding the maximum plasma concentration (C_max_ = 25 nM) (30) (**Figure 4C**). However, based on GR, everolimus effectively inhibited growth of NEC-01013, with values approaching zero in the nanomolar range (**Figure 4D**). The effect of everolimus at 25 nM (C_max_) was significantly stronger in NEC-01013 compared to NEC-01010 and NEC-02002 (**Figure 4E**). In summary, in this limited set of patient derived-organoids, organoids from the responding tumor showed higher sensitivity to both cisplatin and everolimus than organoids from the patients without an objective response.

**Table 4:** Cytotoxicity of cisplatin on NEC organoid lines.

	IC_50_ (µg/ml)^7^	GR_0_ (µg/ml)
NEC-01010^8^	1.17 ± 0.29 (n.s.)^9^	5.36 ± 0.69 *^10^
NEC-01013	0.38 ± 0.08	0.90 ± 0.17
NEC-02002	1.15 ± 0.53 (n.s.)	6.23 ± 1.21*

^7^IC50 and GR0 are given as absolute values with mean ± s.e.m. of 4 (NEC-01010) or 3 (NEC-01013 and NEC-02002) independent experiments.

^8^NEC-01010: passage (P) 6-14; NEC-01013: P10-19; NEC-02002: P6-16.

^9^n.s. indicates non-significant difference compared to NEC-01013.

^10^*indicates significant difference of GR0 compared to NEC-01013 (NEC-01010: p = 0.0089; NEC-02002: p = 0.0050; (ANOVA with Dunnett’s multiple comparisons test).

## Discussion

Here, we describe the establishment and characterization of three novel GEP-NEC organoid lines. Organoid culture may expand the current scientific toolbox to study this rare disease.

Organoids could be classified as neuroendocrine based on the expression of neuroendocrine markers (synaptophysin, chromogranin and CD56). Two organoid lines could not be classified as NEC because they lacked expression of these markers. The tumors from which these organoids were derived were heterogeneous, containing both synaptophysin-positive and -negative tumor cells. Absence of synaptophysin-positive organoids could indicate selective outgrowth of non-neuroendocrine tumor cells or loss of synaptophysin expression during organoid culture. Optimization of media formulation to promote selective outgrowth of neuroendocrine cells could increase the purity of NEC organoid cultures.

Organoid sensitivity to cisplatin and everolimus mirrored patient response in this limited set. Such an association is reassuring, and others have seen correlations between patient response and organoid drug sensitivity (31–33). The predictive value of organoid drug response differs between treatment regimens (32) and studies with larger cohorts are required to determine the validity of organoid response to cisplatin and everolimus as a predictor of clinical response.

Our current culture success rate is 16% (or 10% if only considering organoids confidently classified as NEC), which is too low for clinical implementation at the level of individual patients. This establishment rate is lower than for CRC biopsies (71%) (34) but in the same range as for non-small lung cell cancer (35, 36) or advanced prostate cancer (37). Higher success rates may be achieved by optimizing the culture medium formulation, which may differ depending on the tissue of origin. In our preliminary analysis, establishment rate was not significantly higher for samples grown in tissue-specific rather than generic NEC medium, but higher numbers are needed to draw definite conclusions. Recently, an organoid biobank of neuroendocrine neoplasms has been established, with a considerably higher establishment rate (14). The difference in culture success rate may be attributable to different medium composition. In this study, we received biopsies, which limits the number of different culture conditions that can be tested and may also have contributed to the low culture success rate. Obtaining more tissue, for example by surgical resection of a tumor, will help to further optimize culture conditions.

Two of the NEC models (NEC-01013 and NEC-02007) were derived from metastases and one (NEC-02002) from a primary tumor. Establishment of larger numbers of organoid models from both primary tumors and metastases could serve to evaluate how biological behavior and drug sensitivity changes throughout NEC progression.

NEC organoid models may also be valuable beyond approaches for individual patients. Tumor organoids can help prioritize drug candidates based on genetic or other biological features. Mutations were detected in several DNA repair genes, including those involved in homologous recombination (*RAD50*, *BRIP1*, *RECQL4*). Organoids could be used to evaluate whether drugs targeting DNA repair mechanisms (e.g. PARP inhibitors) are effective in GEP-NEC. We also noted very extensive chromosomal abnormalities in NEC-02002. A high number of copy number alterations has been described previously in GEP-NEC (38). Targeting aneuploidy and/or chromosomal instability (CIN) may present other therapeutic options, such as therapies aimed at increasing proteotoxic stress, or increasing CIN by combining spindle checkpoint inhibitors with taxanes (39) We also identified a *BRAF^V600E^* mutation in the colonic NEC-02002. *BRAF* mutations have been found in 5%–59% of colorectal NEC (40) and two recent case reports describe three colorectal NEC patients that responded to combined BRAF/MEK inhibition (41, 42). NEC organoids could be used for future drug discovery studies.

In conclusion, we have generated three NEC organoid lines that may serve as preclinical models to further the understanding of the biology of NEC. Our approach may also be of value for other rare diseases.

## Data Availability Statement

Requests for sequencing and other data, and for sharing of organoid lines and other resources should be directed to the corresponding author, EV (e.voest@nki.nl) and will be evaluated by the Institutional Review Board of the Netherlands Cancer Institute (IRB@nki.nl). The informed consent signed by patients participating in this study limits the deposition of DNA sequencing data in publicly available repositories. Producer cell lines for Wnt-3a, R-spondinand Noggin-CM were obtained under a material transfer agreement (MTA). Requests for these lines should be directed to the relevant institutions [Hubrecht Institute for Wnt-3a and Noggin, and Calvin Kuo (Stanford) for R-spondin producer lines].

## Ethics Statement

The studies involving human participants were reviewed and approved by the Medical Ethical Committee of the Netherlands Cancer Institute – Antoni van Leeuwenhoek. The patients/participants provided their written informed consent to participate in this study.

## Author Contributions

KD: study concept and design, acquisition of data, analysis and interpretation of data, drafting of the manuscript, statistical analysis. JB: study concept and design, acquisition of pathology data, analysis and interpretation of data, critical revision of manuscript. FW: study concept and design. JH and AV: analysis of sequencing data. SK: processing of tumor tissue. DP: immunohistochemistry. FE and D-JG: patient inclusion and clinical aspects. MT: study concept and design, patient inclusion and clinical aspects, critical revision of manuscript, obtained funding, study supervision. EV: study concept and design, obtained funding, critical revision of manuscript, study supervision. All authors contributed to the article and approved the submitted version.

## Funding

This work was supported by the NWO Gravitation Program (grant number NWO 2012-2022) (to EV on behalf of CancerGenomics.nl), and Ammodo foundation (to MT).

## Conflict of Interest

The authors declare that the research was conducted in the absence of any commercial or financial relationships that could be construed as a potential conflict of interest.
