# Supplementary material for: Patient-Derived Organoid Models of Human Neuroendocrine Carcinoma
Source: Front Endocrinol (Lausanne). 2021 Mar 11;12:627819. doi: 10.3389/fendo.2021.627819 (PMC7991829; doi:10.3389/fendo.2021.627819)
Supplement: Supplementary file 1 [file DataSheet_1.docx]

Supplementary Material

# Supplementary Figures and Tables

## Supplementary Figures

**Supplementary Figure 1. Location of primary tumor and biopsied lesion. (a)** Location of primary tumor from GEP-NEC tumors. Amp. Vater: Ampulla of Vater. **(b)** Location of biopsied lesion of metastasis or inoperable primary tumor.


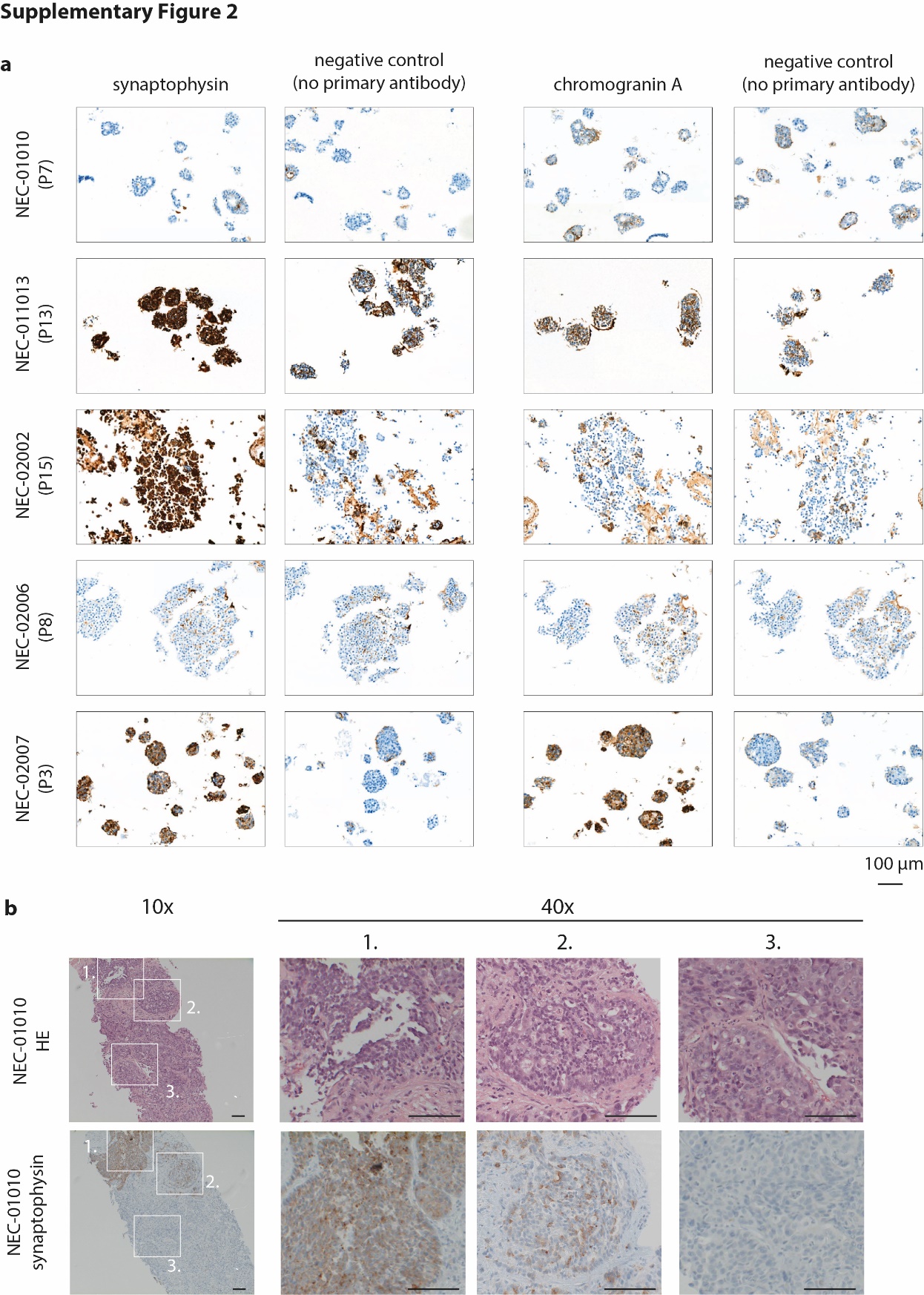


**Supplementary Figure 2. H&E and IHC of original tumors. (a)** Immunostainings for synaptophysin and chromogranin and negative controls. P7 indicates passage 7. **(b)** H&E and immunostains for synaptophysin for different areas of NEC-01010. Boxed areas represent areas with different histomorphology: 1, stereotypical neuroendocrine architecture; 2, more glandular component; 3, intermediate histomorphology. Synaptophysin expression is heterogeneous but expressed in both neuroendocrine and glandular components of the tumor. Scale bar = 100 µm.


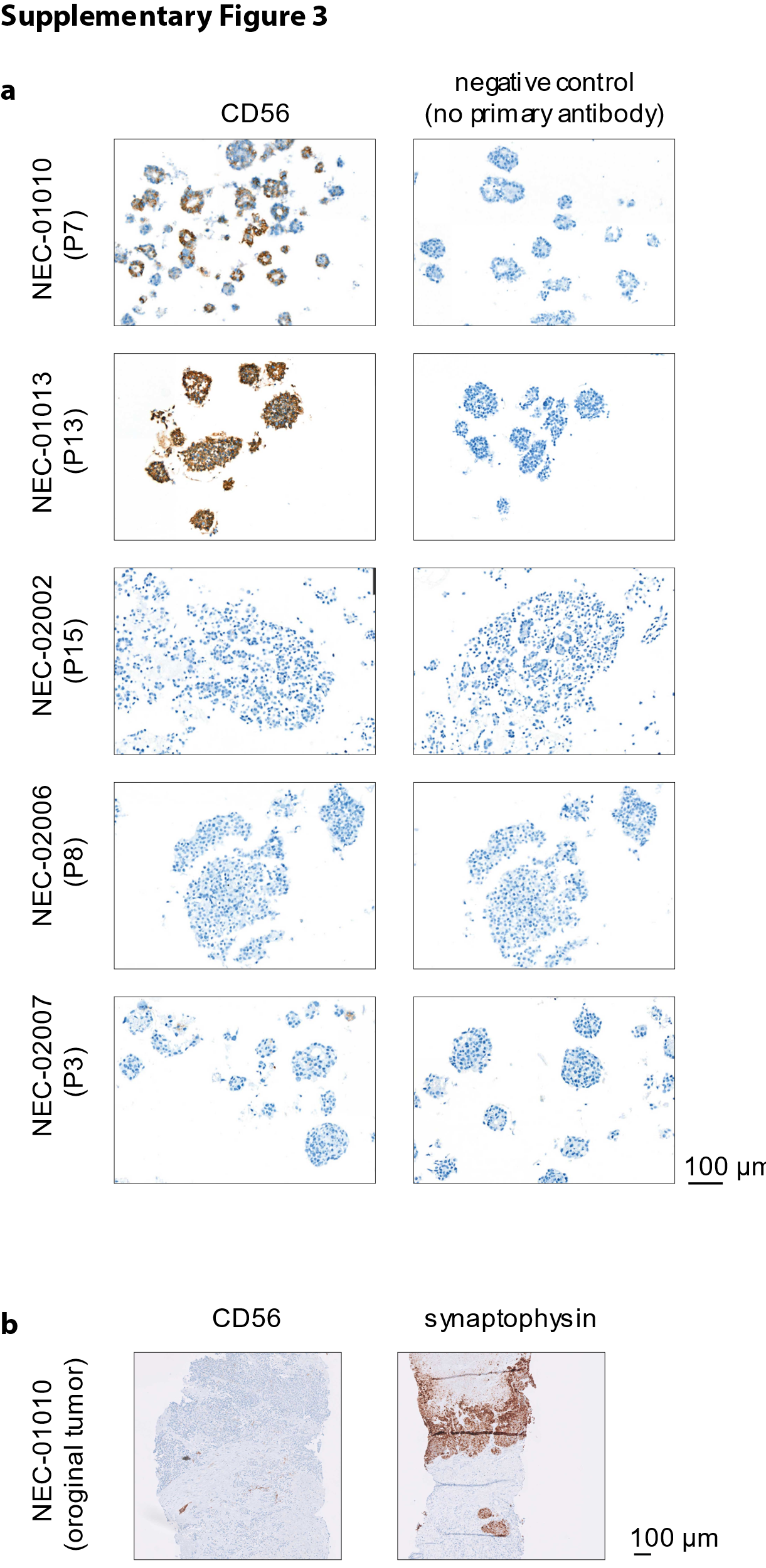


**Supplementary Figure 3. Immunostaining for CD56. (a)** Immunostains of organoids for CD56 and negative controls. P7 indicates passage 7. Scale bar = 100 µm. **(b)** Staining of CD56 and synaptophysin for original tumor from NEC-01010.

## Supplementary Tables

| **Sample** | **Primary tumor** | **Biopsied lesion** | **Organoid success** | **Medium used** | **Reference for medium composition** |
| --- | --- | --- | --- | --- | --- |
| NEC-01001 | Cervix | Liver | N | Generic NEC medium | This paper |
| NEC-01002 | Stomach | Liver | N | Generic NEC medium | This paper |
| NEC-01003 | Stomach | Liver | N | Generic NEC medium | This paper |
| NEC-01004 | Esophagus | Liver | N | Generic NEC medium | This paper |
| NEC-01005 | Unknown | Liver | N | Generic NEC medium | This paper |
| NEC-01006 | Colon | Liver | N | Generic NEC medium | This paper |
| NEC-01007 | Pancreas | Liver | N | Generic NEC medium | This paper |
| NEC-03001 | Rectum | Rectum | N | Generic NEC medium | This paper |
| NEC-03002 | Pancreas | Liver | N | Generic NEC medium | This paper |
| NEC-03003 | Gall bladder | Liver | N | Generic NEC medium | This paper |
| NEC-01008 | Skin | Lymph node | N | Generic NEC medium | This paper |
| NEC-02001 | N/A | Liver | N | Generic NEC medium | This paper |
| NEC-01010 | Amp. Vater | Liver | Y* | Generic NEC medium | This paper |
| NEC-01011 | Unknown | Lymph node | N | Generic NEC medium | This paper |
| NEC-02002 | Colon | Colon | Y | Generic NEC medium | This paper |
| NEC-01012 | Pancreas | Liver | N | Generic NEC medium | This paper |
| NEC-01013 | Stomach | Liver | Y | Generic NEC medium | This paper |
| NEC-01014 | Esophagus | Liver | N | Generic NEC medium | This paper |
| NEC-01017 | Colon | Liver | N | Generic NEC medium | This paper |
| NEC-01018 | Pancreas | Liver | N | Generic NEC medium | This paper |
| NEC-01019 | Cervix | Liver | N | Generic NEC medium | This paper |
| NEC-01020 | Esophagus | Liver | N | Generic NEC medium | This paper |
| NEC-03004 | Pancreas | Liver | N | Pancreas | Boj et al. 2015 |
| NEC-02005 | Ovarium | Peritoneum | N | Ovarium | Kopper et al. 2019 |
| NEC-02006 | Colon | Liver | Y* | Normal colon | van de Wetering et al. 2015 |
| NEC-01022 | Axilla (Merkel cell carcinoma) | Retroperitoneum | N | Generic NEC medium | This paper |
| NEC-01023 | Rectum | Liver | N | Generic NEC medium | This paper |
| NEC-2007 | Esophagus | Liver | Y | Stomach | Bartfeld et al. 2015 |
| NEC-01025 | Cervix | Lymph node | N | Ovarium | Kopper et al. 2019 |
| NEC-02008 | Appendix | Liver | N | Normal colon | van de Wetering et al. 2015 |
| NEC-2009 | Colon | Liver | N | Normal colon | van de Wetering et al. 2015 |

**Supplementary Table 1. Patient samples**. Patient samples for which organoid culture was attempted, with location of original tumor as well as the (metastatic) site biopsied for organoid culture. In the first phase of the study, all samples were cultured in generic NEC medium (Materials and Methods). In the second phase of the study, where possible, samples were cultured in tissue-specific medium. References for the composition of tissue-specific media are provided.
* We successfully established organoids for NEC-01010 and NEC-02006, but do not classify these as NEC organoids given absence of synaptophysin expression.
